# *Notes From the Field:* Dispensing of Oral Antiviral Drugs for Treatment of COVID-19 by Zip Code–Level Social Vulnerability — United States, December 23, 2021–August 28, 2022

**DOI:** 10.15585/mmwr.mm7143a3

**Published:** 2022-10-28

**Authors:** Meg Sullivan, Cria G. Perrine, Jimmy Kelleher, Om Kanwar, Sachiko Kuwabara, Kelly Bennett, Brendan R. Jackson, Pragna Patel, Meghan E. Pennini

**Affiliations:** ^1^Administration for Strategic Preparedness and Response, U.S. Department of Health and Human Services, Washington, DC; ^2^CDC COVID-19 Emergency Response Team; ^3^Palantir Technologies, Palo Alto, California.

Equitable access to COVID-19 therapeutics is a critical aspect of the distribution program led by the U.S. Department of Health and Human Services (HHS).* Two oral antiviral products, nirmatrelvir/ritonavir (Paxlovid)^†^ and molnupiravir (Lagevrio),^§^ received emergency use authorization (EUA) from the Food and Drug Administration (FDA) in December 2021, to reduce the risk for COVID-19–associated hospitalization and death for those patients with mild to moderate COVID-19 who are at higher risk for severe illness (*1*,*2*). HHS has been distributing these medications at no cost to recipients since their authorization. Data collected from provider sites during December 23, 2021–May 21, 2022, indicated substantial disparities in the population-adjusted dispensing rates in high social vulnerability (high-vulnerability) zip codes compared with those in medium- and low-vulnerability zip codes (*3*). Specifically, dispensing rates for the 4-week period during April 24–May 21, 2022, were 122 per 100,000 residents (19% of overall population-adjusted dispensing rates) in high-vulnerability zip codes compared with 247 (42%) in medium-vulnerability and 274 (39%) in low-vulnerability zip codes. This report provides an updated analysis of dispensing rates by zip code–level social vulnerability and highlights important intervention strategies.

Data on dispensed numbers of COVID-19 oral antiviral treatment courses are obtained at regular intervals (at least twice per week) from each provider site receiving medications. The HHS Health Partner Ordering Portal is used by oral antiviral provider partners, including those in all U.S. states and jurisdictions, the Federal Retail Pharmacy Therapeutics Program, and Federal entities (e.g., Indian Health Service, Federal Bureau of Prisons, and the U.S. Department of Defense), to order oral antivirals at no cost and to report inventory and product use.^¶^ Total courses of Paxlovid and Lagevrio dispensed were analyzed by week and zip-code social vulnerability level, using the zip code of the dispensing site. Zip codes were classified as having low, medium, or high social vulnerability, using the same methodology that was used in a previous report**^,^^††^ (*3*).This activity was reviewed by CDC and was conducted consistent with applicable federal law and CDC policy.^§§^

Overall dispensing of oral antivirals increased 57%, from 643 per 100,000 persons during April 24–May 21, 2022, to 1,012 during July 31–August 28. Compared with data collected during April 24–May 21, dispensing during the most recent 4-week period (July 31–August 28) increased to 315 per 100,000 persons (31% of overall population-adjusted dispensing rates) in high vulnerability zip codes, 367 (36%) in medium-vulnerability zip codes, and 331 (33%) in low-vulnerability zip codes (Figure), representing increases in dispensing rates of 159%, 48%, and 21%, respectively. These data indicate a narrowing of the dispensing gap among high-vulnerability and medium-and low-vulnerability zip codes; at the same time, overall dispensing increased. 

**FIGURE:**
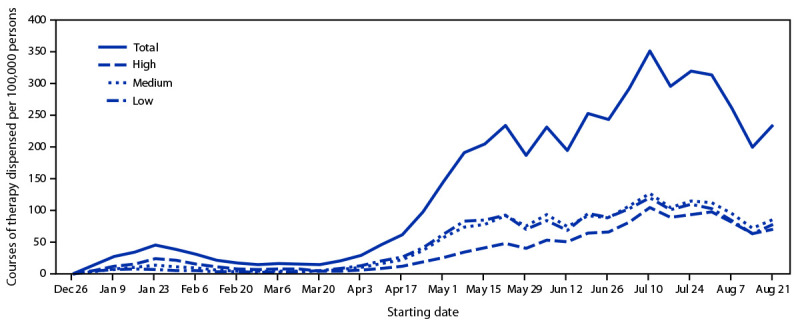
Courses of oral antiviral COVID-19 therapy dispensed per 100,000 persons, by zip code–level social vulnerability — United States, December 23, 2021–August 28, 2022* * Oral antivirals included nirmatrelvir/ritonavir (Paxlovid) (www.fda.gov/media/155050/download) and molnupiravir (Lagevrio) (www.fda.gov/media/155054/download). Zip codes were classified as having low, medium, or high social vulnerability based on ranking within the lower, middle, and upper tertiles of the Equitable Distribution Index score.

HHS worked closely with states and territories to improve equitable dispensing, with a focus on increasing education and awareness^¶¶^ and enhancing distribution efforts, including COVID-19–focused teleprescribing programs, mobile test-to-treat sites, prepositioning of oral antivirals at provider sites in high-vulnerability zip codes, and increased distribution to federally qualified health centers. These efforts were designed to reduce barriers to access by making it easier to satisfy the requirements necessary to receive a clinical assessment to obtain a prescription and begin oral antiviral medication within 5 days of symptom onset. Although both oral antivirals continue to be prescribed primarily by physicians or advanced practice providers, on July 6, 2022, FDA updated the Paxlovid EUA to allow state-licensed pharmacists to prescribe it for an individual patient under certain conditions, including having sufficient data on the patient’s medical history and use of other medications.*** However, pharmacy prescribing of Paxlovid has not yet been widely implemented because of these limitations on prescribing and unclear reimbursement structure for pharmacists to prescribe. Increased dispensing in high-vulnerability zip codes represents valuable progress toward improving access to COVID-19 medications among populations disproportionately affected by the pandemic; however, additional work is needed. Limitations of this analysis include reliance on sites’ self-reported dispensing data, lack of patient-specific data (including demographic and clinical outcome data), and lack of correlative analysis of COVID-19 incidence to overall dispensing rates in specific zip codes.

The COVID-19 therapeutics program represents the largest scale HHS distribution of antivirals, with approximately 16 million COVID-19 treatment courses delivered through August 2022. Ensuring equitable access to antivirals is essential to improving patient outcomes.

## Footnotes

*
https://aspr.hhs.gov/COVID-19/Therapeutics

†
www.fda.gov/media/155050/download

§
www.fda.gov/media/155054/download

¶
https://aspr.hhs.gov/COVID-19/Therapeutics/Distribution/Pages/process-for-ordering.aspx

** Zip code–level social vulnerability was based on the Equitable Distribution Index (EDI) score, used by the federal COVID-19 response because zip code–level data offer a more detailed characterization of population vulnerability than do county level–data and provide sufficient geographic granularity to accomplish operational goals not achievable using U.S. Census Bureau tract–level data. To map U.S. Census Bureau tracts to zip codes, EDI uses a crosswalk file published by the U.S. Department of Housing and Urban Development (https://www.huduser.gov/portal/datasets/usps_crosswalk.html). EDI is not generated for zip codes in which any of the 15 indicators are suppressed within the American Community Survey. This represents <1% of all zip codes mapped to U.S. Census Bureau tract data. https://www.census.gov/programs-surveys/acs

†† Similar to the CDC Social Vulnerability Index (https://www.atsdr.cdc.gov/placeandhealth/svi/index.html), which produces county-level and U.S. Census Bureau tract–level estimates of social vulnerability, EDI uses 15 indicators categorized into four themes: 1) socioeconomic status, 2) household composition and disability, 3) racial and ethnic minority status and language, and 4) housing type and transportation. EDI includes all 15 indicators as a composite measure, and a final score is ranked from lowest (0) to highest (1) vulnerability. A percent rank function is used, such that an equal number of geographic components are in each percentile of the index.

§§ 45 C.F.R. part 46.102(l)(2), 21 C.F.R. part 56; 42 U.S.C. Sect. 241(d); 5 U.S.C. Sect. 552a; 44 U.S.C. Sect. 3501 et seq.

¶¶ Outreach and awareness efforts have included augmentation of direct messaging to communities and partnerships with community-based organizations, collaboration with professional medical associations, development of focused COVID-19 therapeutics webinars and clinical decision toolkits, dissemination of Health Alert Network communications, and National Institutes of Health updates to clinical guidance.

***
https://www.fda.gov/news-events/press-announcements/coronavirus-covid-19-update-fda-authorizes-pharmacists-prescribe-paxlovid-certain-limitations
